# Clinical Benefits, efficacy and tolerability of slowly titrated vortioxetine oral drops solution

**DOI:** 10.1192/j.eurpsy.2024.1130

**Published:** 2024-08-27

**Authors:** A. Fagiolini, A. Cuomo, G. Barillà, M. Cattolico, D. Koukouna, E. Mariantoni, S. Pardossi, C. Pierini, M. Pinzi, G. Piumini

**Affiliations:** Molecular Medicine, University of Siena, Siena, Italy

## Abstract

**Introduction:**

Vortioxetine is mainly prescribed as oral tablets, usually starting at 5-10 mg per day, and is well tolerated by most patients. However, some patients may experience side effects, the most common of which is nausea, which occurs in 20.9-31.2% of people treated with doses of 5-20 mg/day (Baldwin et al, J Psychopharmacol. 2016;30:242-52). In some countries, vortioxetine is also available as an oral solution (1 drop = 1 mg), which allows a very slow titration schedule that may improve tolerability.

**Objectives:**

To evaluate whether vortioxetine oral drop solution, started with 1-2 drops (1-2 mg per day) and increased by 1-2 drops per day to 10-20 drops (10-20 mg), is associated with better tolerability and a lower risk of nausea than that observed with oral tablets started with 5-10 mg per day, while maintaining efficacy. To provide pilot data for the design of a multicentre, prospective study.

**Methods:**

Retrospective, single-centre, observational study. Participants were 58 consecutive patients (mean age 45 + 17 years, 55.2% female) treated with vortioxetine for a depressive episode. Vortioxetine was initiated and titrated up to 1 drop (1 mg) per day in 58.6% of subjects, and initiated and titrated up to 2 drops in 41.4% of subjects. Tolerability was assessed at all visits. CGI and MADRAS scores were recorded at the following time points: T0-baseline, T1=week 1, T2=week 2, T3=week 4, T4=week 8). Comparisons were made using repeated measures ANOVA with Bonferroni correction.

**Results:**

Nausea was reported by 8 subjects (13.8%) at T1, 4 subjects (6.9%) at T2, 1 subject at T3 (1.7%) and none at T4. Other adverse reactions (mainly dizziness, pruritus/itching, vomiting, diarrhoea, and xerostomia) were reported by a total of 6 subjects (10.3%) at T1, none at T2 and T3, and 1 subject (1.7%) at T4. The maximum dose administered was 20 mg in 75.9% of patients. No patients discontinued vortioxetine due to adverse events, but vortioxetine was discontinued prior to T4 (8 weeks of treatment) in 2 subjects due to lack of efficacy. The mean CGI at baseline was 4.3 ±0.8. The mean value decreased to 3.9±0.7 at week 1 and to 3.4±0.6, 2.7±0.6, 1.9±0.5 at weeks 2, 4 and 8, respectively. All differences were statistically significant (p<0.001) compared to baseline. Also from week 2, all scores were statistically significant compared to all previous assessments. The total MADRS score decreased from 28.3±4.6 at baseline to 24.9±4.2, 20.9±4, 16.3±3.6 and 10.9±3 at weeks 2, 4 and 8, respectively. A significant decrease in MADRS total score was observed at each time point (p<0.001) compared to baseline and previous assessments.

**Conclusions:**

Slow titration with vortioxetine oral drop solution was associated with a very low percentage of patients reporting side effects in general, and nausea in particular, and with a relatively rapid improvement in depressive symptoms.

**Disclosure of Interest:**

None Declared

